# A 10-year single-center experience on *Stenotrophomonas maltophilia* resistotyping in Szeged, Hungary

**DOI:** 10.1556/1886.2020.00006

**Published:** 2020-04-23

**Authors:** MÁRIÓ GAJDÁCS, EDIT URBÁN

**Affiliations:** 1 Department of Pharmacodynamics and Biopharmacy, Faculty of Pharmacy, University of Szeged, Eötvös utca 6., 6720, Szeged, Hungary; 2 Department of Public Health, Faculty of Medicine, University of Szeged, Dóm tér 10., 6720, Szeged, Hungary

**Keywords:** *Stenotrophomonas maltophilia*, resistance, resistotype, sulfamethoxazole/trimethoprim, levofloxacin

## Abstract

*Stenotrophomonas maltophilia* is an aerobic, oxidase-negative and catalase-positive bacillus. *S. maltophilia* is a recognized opportunistic pathogen. Due to the advancements in invasive medical procedures, organ transplantation and chemotherapy of malignant illnesses, the relevance of this pathogen increased significantly. The therapy of *S. maltophilia* infections is challenging, as these bacteria show intrinsic resistance to multiple classes of antibiotics, the first-choice drug is sulfamethoxazole/trimethoprim. Our aim was to assess the epidemiology of *S. maltophilia* from various clinical samples and the characterization of resistance-levels and resistotyping of these samples over a long surveillance period. The study included *S. maltophilia* bacterial isolates from blood culture samples, respiratory samples and urine samples and the data for the samples, received between January 2008 until December 2017, a total of 817 *S. maltophilia* isolates were identified (respiratory samples *n =* 579, 70.9%, blood culture samples *n =* 175, 21.4% and urine samples *n =* 63, 7.7%). Levofloxacin and colistin-susceptibility rates were the highest (92.2%; *n =* 753), followed by tigecycline (90.5%, *n =* 739), the first-line agent sulfamethoxazole/trimethoprim (87.4%, *n =* 714), while phenotypic resistance rate was highest for amikacin (72.5% of isolates were resistant, *n =* 592). The clinical problem of sulfamethoxazole/trimethoprim-resistance is a complex issue, because there is no guideline available for the therapy of these infections.

## INTRODUCTION

Antimicrobial resistance (AMR) in Gram-negative bacteria is a major public health concern, severely limiting therapeutic options in clinical settings [1]. While the emergence of plasmidmediated resistance to extended-spectrum cephalosporins (due to AmpC- and extended-spectrum-*β*-lactamases) [2, 3], carbapenems (due to serine- and metallo-*β*-lactamases) [4], and colistin in the members of the Enterobacterales order (predominantly in *Klebsiella pneumoniae)* has taken center-stage in the last few years [5], the clinical problem of infections due to drug resistant non-fermenting Gram-negative bacteria (NFGNB) has been recognized since the beginning of the 21st century [6]. NFGNB are a taxonomically-heterogenous group, including (in decreasing frequency of isolation) *Pseudomonas aeruginosa, Acinetobacter* spp., *Stenotrophomonas maltophilia, Burkholderia cepacia* complex, *Elizabethkingia meningoseptica, Sphingomonas paucimobilis, Alcaligenes faecalis, Achromobacter xylosoxidans* and *Chryseobacterium indologenes* among others [7]. All NFGNB are characterized by their ubiquitous nature in aquatic environments and in the soil (frequently associated with plants); due to their adaptability and tenacity, they are also important nosocomial pathogens, found in ventilator machines and other equipments used for invasive procedures, in addition to water taps, humidifiers or mattress covers in hospital wards [7, 8].

*S. maltophilia* (previously *Xanthomonas maltophilia*) is an aerobic, oxidase-negative and catalase-positive bacillus, which is the principal human pathogen of the genus, currently consisting of 16 different species [9]. In publications before the 1980’s, *S. maltophilia* was reported as an infrequently isolated microorganism from clinical samples, mostly from hospital-acquired infections. Nevertheless, due to the advancements in invasive medical procedures, organ transplantation and chemotherapy of malignant illnesses, the relevance of this pathogen increased significantly since the 2000’s (in correlation with the increased number of patients at risk to develop infections by bacteria with low virulence) [10]. *S. maltophilia* is a recognized opportunistic pathogen. The incidence of *S. maltophilia* infections in nosocomial settings is reported to be around 7–38 cases/10,000 discharges, and it is a frequent cause of outbreaks at intensive care units; in addition, increasing amount of reports highlight the role of these bacteria in community-acquired infections as well [11, 12]. The main clinical manifestations of *S. maltophilia* infections are respiratory infections (i.e., tracheobronchitis) and bacteremia, however, infections from almost all anatomical regions have been described (e.g., meningitis, skin and soft tissue infections, genitourinary infections) [13, 14]. The crude mortality rate for invasive *S. maltophilia* infections is quite high, especially if the patients receive inappropriate empiric therapy: 20–60% in case of bacteremia/sepsis and 20–70% in case of pneumonia [15, 16]. The colonization of cystic fibrosis patients with *S. maltophilia* has also been extensively described, often leading to more frequent exacerbations and worse outcomes [17].

The therapy of *S. maltophilia* infections is challenging, as these bacteria show intrinsic resistance to multiple classes of antibiotics [9]. From a clinical perspective, resistance against *β*-lactam antibiotics (most notably, the carbapenem group) is a major concern; this is conferred by two zinc-dependent, chromosomally mediated *β*-lactamases (L1 and L2) [14, 18]. In addition, a resistant phenotype may be expressed through a multitude of other mechanisms, e.g., lipopolysaccharide-changes or modifying enzymes for aminoglycosides, or through the over-expression of energy-dependent efflux pumps (e.g., SmeDEF, SmeVWX, SmeYZ), affecting susceptibility to several drugs [19]. Based on clinical experiences and current recommendations, the first-choice drug for the therapy of *S. maltophilia* infections is sulfamethoxazole/trimethoprim (or co-trimoxazole; 15 mg kg^–1^ day^–1^) [12, 14]. Additionally, a recent meta-analysis has concluded that the use of levofloxacin in these infections is non-inferior to sulfamethoxazole/trimethoprim [20]. Nonetheless, in certain clinical situations (hypersensitivity to the drug, vulnerable patient population to fluoroquinolones) and in case of resistance to these agents, alternative drugs must be considered, usually in combination: these antibiotics include the tetracyclines (doxycycline, minocycline, and tigecycline), some remaining *β*-lactams with retained activity (ticarcillin/clavulanate, ceftazidime), colistin, rifampin and chloramphenicol [9, 12, 14]. Resistance to the first-line agent sulfamethoxazole/trimethoprim is around 2–10% in Western Europe and in the US, however, resistance rates as high as 30–48% were reported from the Far East (China, Taiwan) [21]; resistance levels are generally higher in colonizer strains from cystic fibrosis patients (20–80%) [22]. Multidrug resistant (MDR) and extensively drug resistant (XDR) strains of *S. maltophilia* are concerning from both therapeutic and infection control perspectives, thus, the World Health Organization listed this pathogen as a “priority pathogen” for pharmaceutical companies to incentivize development of novel antibiotics [23].

Several surveillance studies have been published on the epidemiology of this pathogen, however, these epidemiological trends and resistance levels vary greatly in each hospital and geographical region; while the knowledge of local data is necessary to reflect on the regional/national situation and to allow for the appropriate choice of therapy [24]. In the present study, our aim was to assess the epidemiology of *S. maltophilia* from various clinical samples and the characterization of resistance-levels in these samples over a long surveillance period in a tertiary-care teaching hospital in Southern Hungary.

## MATERIALS AND METHODS

### Clinical center

The present retrospective microbiological study was carried out at the Albert Szent-Györgyi Clinical Center, a tertiary-care teaching hospital in Szeged, Hungary. The study included *S. maltophilia* bacterial isolates from blood culture samples, respiratory samples and urine samples and the data for the samples from all outpatient Clinics and impatient departments, corresponding to the time period between January 2008 until December 2017. Bacterial isolates were considered separate if they were detected more than 14 days apart, or *S. maltophilia* isolates with different antibiotic susceptibilities were isolated [12]. Isolates collected for surveillance/infection control purposes from hospital environments were excluded from the analysis.

### Sample processing and bacterial identification

Blood culture samples, respiratory samples and urine samples were processed in the Institute in accordance with international guidelines in routine bacteriology. Between 2008 and 2012, the BD Bactec (Beckton Dickinson, Franklin Lakes, NJ, USA) automated blood culture system was employed in the Institute, while from 2013 onwards, the BacT/ALERT 3D (bioMérieux, Marcy-l’Étoile, France) detection system was utilized. Blood culture bottles were incubated for 5 days (21 days, if endocarditis was suspected). Samples were cultured on blood agar, chocolate agar, eosinemethylene blue or UriSelect agar (in case of urine samples) plates (agar plates purchased from Bio-Rad, Berkeley, CA, USA). Culture plates were incubated at 37 °C for 24–48 h, aerobically. Between 2008 and 2012, phenotypic methods and VITEK 2 Compact ID/AST (bioMérieux, Marcy-l’Étoile, France) were used, while following 2012, matrix-assisted laser desorption/ionization time-of-flight mass spectrometry (MALDI-TOF MS; Bruker Daltonics, Bremen, Germany) was introduced to the diagnostic workflow of the laboratory. Sample preparation methods and the technical specifications for MALDI-TOF MS measurements were described elsewhere [25].

### Antimicrobial susceptibility testing, resistotyping

Susceptibility-testing of *S. maltophilia* isolates were carried out using the following methods and protocols: i) sulfamethoxazole/trimethoprim susceptibility testing was carried out using E-tests (Liofilchem, Abruzzo, Italy) on Mueller-Hinton agar plates, based on EUCAST breakpoints (http://www.eucast.org; MIC ≤ 4 mg L^–1^ reported as susceptible); ii) levofloxacin susceptibility testing was performed using E-tests (Liofilchem, Abruzzo, Italy) on Mueller-Hinton agar plates, based on CLSI breakpoints (MIC ≤ 2 mg L^–1^ reported as susceptible); iii) amikacin susceptibility testing was based on a *P. aeruginosa-specific* breakpoint using E-tests (Liofilchem, Abruzzo, Italy) on Mueller-Hinton agar plates (MIC ≤ 16 mg L^–1^ reported as susceptible) [12]; iv) colistin susceptibility testing was based on a *P. aeruginosa-specific* breakpoint using broth microdilution in cation-adjusted Mueller-Hinton broth (MERLIN Diagnostika, Bornheim-Hersel, Germany) (MIC ≤ 4 mg L^–1^ reported as susceptible) [12]; v) tigecycline susceptibility-testing was carried out using E-tests (Liofilchem, Abruzzo, Italy) on Mueller-Hinton agar plates, the interpretation of results was carried out using non-species specific (NSS) breakpoints (MIC ≤ 0.25 mg L^–1^ reported as susceptible) [12]. Classification of the isolates as a multidrug resistant (MDR) or extensively drug resistant (XDR) was based on the EUCAST Expert Rules [26]. Resistotypes from the respective susceptibility-results were defined based on criteria described previously [27, 28]. *Staphylococcus aureus* ATCC 29213, *Enterococcus faecalis* ATCC 29212, *Escherichia coli* ATCC 25922, *P. aeruginosa* ATCC 27853 and *S. maltophilia* ATCC 13637 were used as quality control strains.

### Statistical analysis

Descriptive statistical analysis was performed using Microsoft Excel 2013 (Redmond, WA, Microsoft Corp.). Additional statistical analyses were performed with SPSS software version 24 (IBM SPSS Statistics for Windows 24.0, IBM Corp. Armonk, NY, USA), using the χ^2^-test and two-sample-test (isolation frequency and resistance trends). *P* values <0.05 were considered statistically significant.

### Ethics

The study was deemed exempt from ethics review by the Institutional Review Board of the University of Szeged and informed consent was not required, as patient data was not collected and data anonymity was maintained.

## RESULTS

### Isolation frequency of S. *maltophilia*

During the 10-year period, a total of 817 *S. maltophilia* isolates were identified (81.7 ± 31.0 year^–1^, highest in 2015, lowest in 2008). The distribution of the samples of origin was the following: respiratory samples *n =* 579 (70.9%), blood culture samples *n =* 175 (21.4%) and urine samples *n =* 63 (7.7%). A pronounced increase was observed in the isolation frequency of *S. maltophilia* isolates between two 5-year periods of the study (2008–2012: *n =* 263, 2013–2017: *n =* 554; *P =* 0.0011). The majority of isolates originated from samples sent from inpatients *(n =* 694, 84.9%). Isolates originated from the Intensive Care Units (ICUs; 41.9%; *n =* 334), Department of Internal Medicine (29.5%; *n =* 241), Department of Pediatrics (10.1%; *n =* 74), Department of Otorhinolaryngology and Head-Neck Surgery (6.4%; *n =* 52), Department of Oncology (4.7%; *n =* 38), Department of Surgery (4.0%; *n =* 33), Department of Neurology (1.5%; *n =* 13) and others (n = 17; 1.8%).

### Antibiotic resistance and resistotypes of *S. maltophilia*

Out of the tested antibiotics, levofloxacin and colistin-susceptibility rates were the highest (92.2%; *n =* 753), followed by tigecycline (90.5%, *n =* 739), the first-line agent sulfamethoxazole/trimethoprim (87.4%, *n =* 714), while phenotypic resistance was most frequently observed for amikacin (72.5% of isolates were resistant, *n =* 592). 24.1% (n = 197) of isolates were fully susceptible to all five tested agents. Resistance to sulfamethoxazole/trimethoprim occurred more frequently in the second half of the study period (66 vs. 37; *P =* 0.047), while such trends were not observed for the other antibiotics. Similarly, sulfamethoxazole/trimethoprimresistance was also detected more frequently from inpatient samples *(P =* 0.004). MIC ranges for the respective antibiotics were the **f**ollowing: MIC_sulfamethoxazole/trimethoprim_ = 0.064–32 mg L^–1^, MIC_levofloxacin_ = 0.25–16 mg L^–1^, MIC_amikacin_ = 2–512 mg L^–1^, MIC_colistin_ = 0.5–512 mg L^–1^ and MIC_tigecycline_ *=* 0.064–8 mg L^–1^.

The distribution of isolates into various resistotypes is shown in Table 1.; *Type 0* represents fully-susceptible isolates (24.1%), *Type I* includes isolates resistant to amikacin or tigecycline only (65.4%), while *Type II* (1.8%) and *Type III* (4.0%) introduces resistance to sulfamethoxazole/trimethoprim and levofloxacin, respectively. *Type IV* (7.0%) represents resistance to three, while *Type V* (1.0%) represents resistance to four individual antibiotics. *Type VI* (2.2%) encompasses strains showing resistance to all tested agents. Based on EUCAST Expert Rules, isolates in Type IV and V categories also represent MDR *S. maltophilia* isolates, while isolates in the Type VI category should be considered XDR.

## DISCUSSION

The aim of our present study was to characterize the resistance levels of *S. maltophilia* in a tertiary-care teaching hospital in the southern region of Hungary over a long surveillance period using phenotypic methods. *S. maltophilia* is an emerging, opportunistic pathogen with low levels of invasiveness, mainly affecting severely debilitated patients [29]. The following risk groups have been identified based on the literature: ICU patients or patient with a long hospital stay, extensive surgeries, immunosuppressive therapy or acquired immunosuppression (e.g., HIV-infection, severe neutropenia), mechanical ventilation, dialysis, patients with chronic illnesses (e.g., diabetes, respiratory disorders) or cancer, or a developmental abnormality [30]. Prevention of *S. maltophilia* acquisition and infection is very important from an infection control point-of-view, in addition to controlling antibiotic consumption for reducing the emergence of resistant strains [31]. Extensive use of carbapenems (both on a patient-level and institutional-level) has also been described as a potential risk factor for these infections (due to the selection pressure) [32]. The gastrointestinal tract, infected central venous catheters and the colonized/infected lungs were described as sources of infection, leading to invasive disease [33]. Due to its limited invasiveness, *S. maltophilia* must somehow bypass natural host defenses to cause illness; nonetheless, virulence factors, such as biofilm-formation (important for survival on abiotic surfaces), a positively charged cell surface and fimbriae are all considered important during the pathogenesis of these infections [34]. Previously it was hypothesized that *S. maltophili*a infections are characterized by the lack of an inflammatory response, however, this dogma has been recently challenged in a murine model, where it was shown that airway epithelial cells and macrophages react with an increased expression of IL-8 and TNF-α [35].

Empiric therapy of *S. maltophilia* infections is sulfamethoxazole/trimethoprim, combined with levofloxacin or ticarcillin/clavulanate (if available); the therapeutic protocol should be revised after the susceptibility results are available [1, 2, 12, 14]. Resistance rates to sulfamethoxazole/trimethoprim (12.6%) was higher than the range of resistance in Western European countries (2–10%), although outlier countries with higher resistance (e.g., Spain: 25–27%, Turkey: 10–15%) have already been noted [36, 37]. In contrast, the low level of levofloxacin resistance is an advantageous development, as it seems that there is no relevant difference in the clinical efficacy of these two drugs [20]. The relevance of the other three tested agents in clinical situations is harder to ascertain, as there are no evidence or clinical trials correlating their efficacy in the therapy of *S. maltophilia* infections [38]. In addition (as demonstrated in the Methods section), there are also contradictory information regarding susceptibility-testing method for these bacteria: based on EUCAST, disk diffusion is only available for sulfamethoxazole/trimethoprim, while CLSI offers disk diffusion testing breakpoints for levofloxacin and minocycline as well [12, 14, 39]. Some drugs, not even MIC breakpoints are available (thus, clinical microbiologists should not interpret them as susceptible or resistant for the treating physicians), as the pharmacokinetic/pharmacodynamic attributes, outcomes and antimicrobial efficacy of these antibiotics have not been characterized in relation with *S. maltophilia* infections [12, 14, 39]. The clinical problem of sulfamethoxazole/trimethoprim-resistance (mediated by the *sul1-sul3* genes) is a complex issue. Because there is no guideline available for the therapy of these infections, clinicians often act upon national and/or institutional guidelines [40]. The development of guidelines would require reliable data from multiple clinical trials utilizing antibiotics other than sulfamethoxazole/trimethoprim, with clearly-defined case definitions and clinical endpoints; unfortunately, such data is currently not available [39, 40].

The definition of resistotyping is the grouping of bacterial isolates by resistance patterns to a set of arbitrarily chosen antibiotics that are characteristic to specific strains by phenotypic methods; resistotyping is mainly used for epidemiological purposes [41]. Although data has been generated on the resistance-levels of *S. maltophilia* in other regions of Hungary (where the reported susceptibility to sulfamethoxazole/trimethoprim higher than in the present study [99%], in contrast, susceptibility to levofloxacin [75%], tigecycline [12%] and colistin [9%] were reported to be much lower [39]), resistotyping for this pathogen has not been previously described locally or in any other studies published previously. To highlight their importance, resistotypes may be correlated with clinical-therapeutic decisions: e.g., resistotypes 0, IA and IB are pan-susceptible, or resistant only to ancillary antibiotics, thus, the first-line drug (sulfamethoxazole/trimethoprim alone or in combination) may be used without difficulty, if the underlying conditions or the patient’s medical history allows for it. Resistotypes IIA and IIB are resistant to sulfamethoxazole/trimethoprim, but susceptible to levofloxacin, which is presumably just as clinically-effective as the first-line drug; depending on the age of the patient, this drug may be clinically used alone or in combination (with ticarcillin/clavulanate, ceftazidime or rifampin). Resistotypes IIIA and IIIB are resistant to the tested fluoroquinolone drug, but susceptible to sulfamethoxazole/trimethoprim, corresponding to a similar therapeutic approach like resistotypes 0, IA and IB. Therapy of these infections becomes especially problematic starting from the IVA resistotype all the way onto resistotype VI, where, in addition to resistance against sulfamethoxazole/trimethoprim and/or levofloxacin, the utility of possible secondary antibiotics is also narrowing: resistance to amikacin, tigecycline and colistin means that only very few antibiotics are left for therapy and for most of these agents, clinical evidence of efficacy is limited to case reports [42].

The relevance of amikacin is often questioned, as resistance may quickly develop due to membrane impermeability or alterations in the bacterial LPS, while colistin is considered as one of the last-resort agents, due to its nephrotoxic and neurotoxic adverse events and difficult dosing [43, 44]. Several reports highlight the efficacy of tetracycline-derivatives, especially minocycline as a potential therapeutic alternative for resistant *S. maltophilia* infections, demonstrating high cure rates and advantageous outcomes. However, the adverse effect-profile of these drugs and the low serum concentrations achieved by tigecycline should also be taken into consideration [45]. Besides this, concerns have been raised that the frequent use of minocycline for the therapy of *Acinetobacter calcoaceticus-baumannii* complex may lead to the emergence of resistance in *S. maltophilia* [46].

The following limitations of the study should be noted: i) as the clinical data of the individual patients affected could not be accessed, the correlation between the symptoms and the isolation of *S. maltophilia* is unknown; thus, all true pathogens and colonizers were included in this study; ii) resistance of these isolates were characterized only phenotypically, the genetic nature of these resistance-determinants were not detected using molecular biological methods; iii) minocycline susceptibility-testing was not performed as this drug is not licensed or available in Hungary; iv) referral/selection bias as the clinical center is a tertiary-care, specialized hospital.

## CONCLUSIONS

*S. maltophilia* is an emerging opportunistic pathogen predominantly isolated from blood culture and respiratory tract samples, most often causing bacteremia, tracheobronchitis and soft tissue infections in hospitalized, immunocompromised patients. It is often difficult to distinguish between colonization and true infection, if the bacteria have been isolates from non-sterile body sites, however, the surveillance of colonizers is also relevant as in most cases, these microorganisms will initiate infections in susceptible hosts. The pharmacotherapy of *S. maltophilia* infections is limited by high-level intrinsic resistance, which is often worsened by acquired non-susceptibility. In our study, 87.4 and 90.5% of isolates were susceptible to sulfamethoxazole/trimethoprim and levofloxacin, respectively; in these cases, first-line agents are appropriate for use, bearing in mind the adverse events and contraindications associated with these drugs for specific patient groups. On the other hand, 8.0% were MDR and 2.2% was to be considered XDR strains. Thus, clinical management of infections, where – for whatever reason – none of the first-line agents are available for use, depends on the susceptibility of the pathogen to ancillary agents and the availability of these antibiotics on an institutional/regional/national level. Additionally, more studies are needed to adequately assess the relevance of such antibiotics (i.e., colistin, minocycline, tigecycline, amikacin, rifampin, ticarcillin/clavulanate or ceftazidime) in the management of resistant *S. maltophilia.*

## Figures and Tables

**Table 1.** Distribution of various resistotypes among *S. maltophilia* (2008–2017)

Resistotype	Sulfamethoxazole/trimethoprim	Levofloxacin	Amikacin	Tigecycline	Colistin	Number of isolates
0	S	S	S	S	S	*n =* 197 (24.1%)
I-A	S	S	**R**	S	S	*n =* 489 (59.9%)
I-B	S	S	S	**R**	S	*n =* 45 (5.5%)
II-A	**R**	S	S	S	S	*n =* 10 (1.2%)
III-A	S	**R**	S	S	S	*n =* 20 (2.4%)
III-B	S	**R**	**R**	S	S	*n =* 13 (1.6%)
IV-A	**R**	**R**	**R**	S	S	*n =* 5 (0.6%)
IV-B	**R**	S	**R**	**R**	S	*n =* 11 (1.3%)
IV-C	**R**	S	**R**	S	**R**	*n =* 42 (5.1%)
V-A	**R**	**R**	**R**	**R**	S	*n =* 4 (0.5%)
V-B	**R**	**R**	**R**	S	**R**	*n =* 4 (0.5%)
VI	**R**	**R**	**R**	**R**	**R**	*n =* 18 (2.2%)

S: susceptible; R: resistant.
